# A reproducible method for the expansion of mouse CD8 + T lymphocytes

**DOI:** 10.1016/j.jim.2015.01.004

**Published:** 2015-02

**Authors:** Mark D. Lewis, Evy de Leenheer, Sigal Fishman, Lai Khai Siew, Gideon Gross, F. Susan Wong

**Affiliations:** a Institute of Molecular and Experimental Medicine, School of Medicine, Cardiff University, Cardiff CF14 4XN, UK; b Laboratory of Immunology, MIGAL Galilee Research Institute, Kiryat Shmona 11016, Israel; c Department of Biotechnology, Tel-Hai College, Upper Galilee 12210, Israel

**Keywords:** CD8 + T cells, Activation, Immunotherapy

## Abstract

Murine adoptive CD8 + T-cell immunotherapy studies require the generation of large numbers of high viability CD8 + cells. Here we report a tissue culture protocol for the reliable expansion of CD8 + T-cells derived from murine spleen to give a 20-fold expansion after 4 days in culture. The cells were transfected with an mRNA GFP construct and transferred into NOD mice. GFP positive cells could be detected 7 days after transfer thus confirming that the cells survive and are functional for up to 1 week.

## Highlights

• A technique for reliable expansion of murine CD8 + T cells
• Excellent cell viability
• Allows for electroporation of cells with mRNA constructs and detection of expression up to 7 days *in vivo*

## 1 Introduction

Recent advances in the use of immunotherapy for the treatment of cancers have led to the development of reliable methods for the large-scale expansion of human CD8 + T lymphocytes (Gattinoni et al., 2006; Zhang et al., 2014). Such studies are often preceded by “proof of principle” studies in mice. However there is a lack of reliable methods for expanding large numbers of mouse CD8 + T cells. Here we report a method for the reproducible, large-scale expansion of mouse CD8 + T cells of high viability and their use in transfection and *in vivo* tracking experiments.

## 2 Material and methods

### 2.1 Mice

Non-obese diabetic (NOD) and C57BL/6 mice were used. All procedures were performed in accordance with the protocols approved by the UK Home Office.

### 2.2 CD8 + T cell isolation

CD8 + T cells were isolated from the spleens under aseptic conditions. Individual spleens were homogenized to release splenocytes, in 5 ml RPMI 1640 medium. The cell suspension was centrifuged (5 min at 400 g at room temperature), the supernatant decanted and the cells resuspended in the residual volume (approximately 100 μl). Erythrocytes were lysed briefly in 900 μl sterile water (1–2 s) before the addition of 100 μl 10 × PBS to restore iso-osmolarity and 5 ml serum-free RPMI 1640 medium. The single-cell suspensions from individual spleens were pooled, filtered through a 40 μm cell strainer (Fisher) and counted. CD8 + T cells were isolated using magnetic cell sorting by negative selection (CD8a^+^ T Cell Isolation Kit II, Miltenyi) according to the manufacturer's instructions. Cell samples were taken prior and post-magnetic selection followed by staining with a fixable viability dye (eFluor 506, eBiosciences), anti-CD8-APC (clone 53-6.7, BioLegend), anti-CD4-FITC (clone GK1.5, BD Pharmingen), and anti-CD19-PerCPCy5.5 (clone 1D3, BD Pharmingen) to check sample purity.

### 2.3 Cell culture

T cells were initially plated into 6-well plates (Nunc, Thermo Fisher Scientific) coated with anti-CD3 (clone 2C11, Bio X Cell) and anti-CD28 (clone 37.51, Bio X Cell). The plates were coated with 0.5 μg/ml anti-CD3 and 5 μg/ml anti-CD28 in PBS (5 ml/well) overnight at 4 °C and washed twice with PBS prior to plating cells. The CD8 + T cells were plated (day 0) at 5 × 10^6^ per well in RPMI medium (PAA Laboratories) containing 10% heat-inactivated fetal bovine serum (Sigma) and supplemented to a final concentration with l-glutamine (2 mM), penicillin (50 U/ml), streptomycin (50 μg/ml), 2-mercaptoethanol (50 μM), 1% ITS: insulin (1.7 μM), transferrin (68.8 μM) and sodium selenite (3.9 nM), all from Life Technologies. The cells were cultured for 24 h (37 °C, 5% CO_2,_ day 1), after which IL-7 (recombinant mouse, R & D Systems) and IL-2 (EL-4 culture supernatant, Wong et al., 1996) were added to final concentrations of 0.5 ng/ml and 30 U/ml respectively. The cells were then cultured for a further 24 h (day 2). At this point, the cells were harvested from the wells by resuspending them in the medium using a plastic Pasteur pipette and subcultured by plating at 1 × 10^6^/well into a 6-well plate, fresh medium containing all supplements including IL-7 and IL-2 being added to a final volume of 5 ml/well. The cells were cultured for a further 24 h (day 3), harvested and re-plated at 1 × 10^6^/well as for day 2 and cultured for a further 24 h. Cells were harvested on day 4 for transfection.

### 2.4 Transfection and cell transfer

A GFP construct (GFP in pGEM4Z/GFP/A64 vector; Boczkowski et al., 2000) was used as template DNA for *in vitro* transcription of RNA. mRNA was synthesized from this DNA template with a T7 mScript Standard mRNA Production System (CellScript, Madison, WI, USA; Perez et al., 2014). CD8 + T cells were harvested, pooled and pelleted by centrifugation (400 g for 5 min). The cells were then washed twice with 20 ml Opti-MEM (Life Technologies) by resuspension and centrifugation, before being resuspended in cold Opti-MEM at 10 × 10^6^/200 μl and kept on ice. The cells (10 × 10^6^) were transfected with 5 μg GFP mRNA, by electroporation (BTX Harvard Apparatus ECM830) at 300 V for 1 ms in a precooled 2 mm cuvette (BTX). After transfection, the cells were added to complete medium containing IL2 and IL7 (< 2 × 10^6^ cells/ml) and rested for 3 h at room temperature with periodic mixing to prevent the cells from pelleting. The cells were washed twice in PBS and resuspended at 5 × 10^7^ cells/ml in normal saline. As a control, CD8 + T cells were subjected to the electroporation procedure but with no added mRNA (mock transfection). Transfected cells (GFP and mock transfected) were adoptively transferred into 6-week old NOD mice recipients by injection into the tail vein (10 × 10^6^ cells per mouse). Lymphoid tissues (spleen and pancreatic lymph nodes) were harvested after 1, 3 and 7 days. GFP positive cells were identified by flow cytometry.

### 2.5 Flow cytometry

Cells (1 × 10^6^ per tube) were stained with anti-CD8-PECy7 (clone YTS156.7.7, BioLegend), and with a viability dye (eFluor 506, eBioscience) for studies testing GFP expression. Cells (1 × 10^6^) were stained for cell surface antigens using the following antibody panels: panel 1, anti-CD8-APC (clone 53-6.7, BioLegend), anti-CD103-FITC (clone 2E7, eBioscience), anti-LFA-1/CD11a-PE-Cy7 (clone M17/4, eBioscience), anti-CD25-PE (clone PC61, BioLegend) and with a viability dye (eFluor 780, eBioscience); panel 2, anti-CD8-FITC (clone 53-6.7, BD Bioscience), anti-CTLA-4-Brilliant Violet 421 (clone UC10-4B9, BioLegend), anti-CD127/IL-7Rα-PE (clone SB/199, BD Bioscience), anti-KLRG1-APC (clone 2F1/KLRG1, BioLegend), anti-CD69-PECy7 (clone H1.2F3, BioLegend) and with a viability dye (eFluor 780, eBioscience); and panel 3, anti-CD8-APC (clone 53-6.7 BioLegend) and anti-CD215/IL-15Rα-PE (clone DNT15Ra, eBioscience). Samples for intracellular cytokine staining were treated with monensin (2 μM), PMA (80 nM) and ionomycin (0.8 μM) for 3 h prior to staining. Cells (1 × 10^6^ per tube) were first stained with anti-CD8-FITC (clone 53-6.7, BD Bioscience) and a viability dye (eFluor 506, eBioscience). Following surface marker staining cells were fixed and permeabilized (BD Bioscience, CytoFix/CytoPerm Buffer) and then stained with anti-IL-10-PerCP Cy5.5 (clone JESS-16E3, eBioscience), anti-interferon gamma-APC (clone XMG1.2, BD Bioscience) and anti-TNFα-PE (clone MP6-XT22, BD Bioscience). Samples were run on a FacsCanto II (BD) flow cytometer and data were analyzed using FlowJo software (Tree Star). Isotype controls were run for intracellular cytokine staining.

## 3 Results

**Fig. 1:**
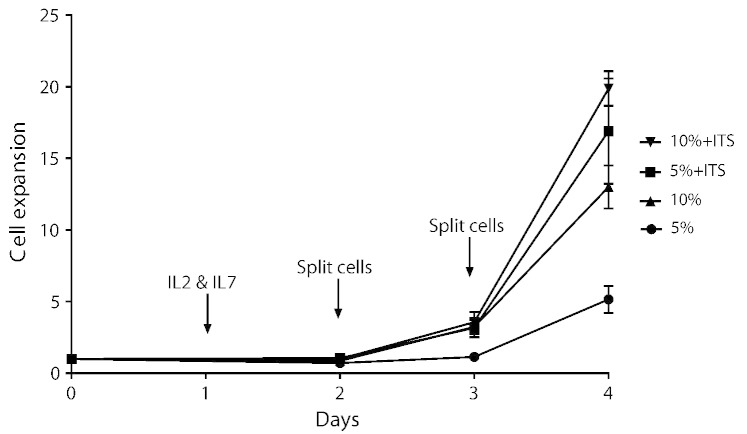
CD8 + T cell expansion with different growth media supplements. Growth rates of CD8 + T cells in RPMI medium with either 5 or 10% FCS and ITS (+). Cell expansion is defined as fold change with respect to day 0, where day 0 is defined as 1. The timings of the addition of cytokines and sub-culture are also indicated. The results are the mean of 4 separate experiments ± SE.

Negative selection of CD8 + cells gave a 91% pure population of CD8 + cells (89%–95.6%, n = 5) with CD4 contamination of 0.21% (0.1%–0.49%, n = 5), B cell contamination of 1.58% (0.38%–3%, n = 5) and very high viability (> 99%). Expansion of negatively selected NOD CD8 + T cells using the protocol described above was improved by increasing the FCS concentration from 5% to 10% and improved further by supplementing the medium with ITS. These comparisons are shown in Table 1 and Fig. 1.

The combination of increasing the FCS to 10% and including ITS in the medium significantly increased the expansion compared to RPMI 5% without ITS (20 fold CD8 + T cell expansion ± 1.2 compared to 5.1 fold CD8 + T cell expansion ± 0.95, p < 0.01, n = 4). The addition of ITS to RPMI 5% increased expansion (16.7 ± 3.8, p < 0.01), as did increasing the FCS to 10% without ITS (13 ± 1.5, p < 0.05) but to a lesser extent than RPMI 10% + ITS. The viability of the cells in all three was increased compared to RPMI 5% (Fig. 1).

In order to look at the effect of the expansion/activation process on the cells, they were stained with markers of T cell activation. Non-expanded NOD spleen cells were also stained as a control. The results are expressed as the percentage of live CD8 + cells that are positive for each marker and are shown in Table 2. As expected, CD25, LFA1, CTLA4, IL-7R and IL-15R were upregulated on the cells and they considerably increased the proportion of IFN-γ and TNF-α producing cells, but IL-10 was not stimulated. As the cells were examined after 4 days of expansion, CD69 was not raised, in keeping with the time course of increase, as it peaks within the first 24–48 h, and KLRG1, a marker of memory cells was not upregulated as the time course was too short for memory generation.

Transfection efficiency of the expanded NOD CD8 + cells was assessed by flow cytometry following transfection with a GFP- expressing mRNA construct by electroporation. Six hours after transfection, > 95% of live cells were GFP positive and after 3 days in culture, > 92% of live cells were still GFP positive as assessed by flow cytometry. This confirmed that this method is able to transfect the cells with high efficiency and that expression of the encoded protein, is rapid and sustained for a period of at least 3 days *in vitro*.

**Fig. 2:**
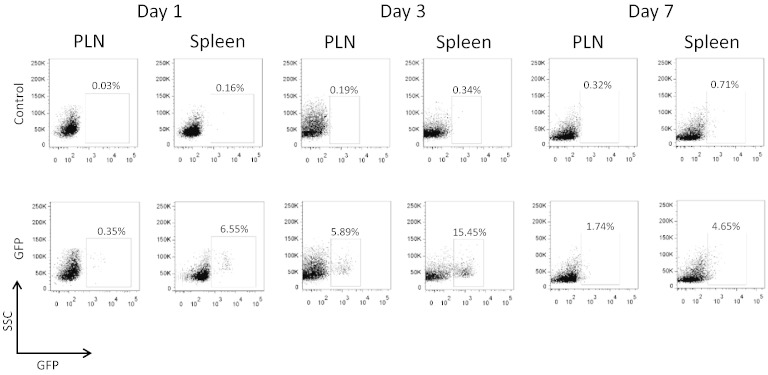
Time course of detection of GFP- transfected cells in spleen and pancreatic lymph node. Flow cytometric plots illustrating gated CD8 + T cells, with percentages indicating GFP-positive CD8 + T cells. These cells were detected in both spleen and pancreatic lymph nodes (PLN) 7 days after adoptive transfer of the transfected cells.

In order to test the suitability of the expanded CD8 + T cells for immunotherapy studies following a transfection procedure, the cells were transfected with the mRNA GFP construct, intravenously injected into NOD mice and lymphoid tissues harvested over a time course. The viability of transfected cells was assessed immediately prior to transfer and routinely found to be in excess of 90%, with no difference between mock or GFP- transfected cells. GFP-positive CD8 + T cells were detected at 1, 3 and 7 days after injection (Table 3 and Fig. 2), confirming that the cells express the construct and are capable of surviving for up to 7 days *in vivo*.

GFP-positive cells were detected in the spleen and PLN of the mice that received GFP transfected cells. At day 1, GFP-positive cells were only detected in the spleen but by day 3, GFP-positive cells could be detected in spleen and PLN, the greatest number being in the spleen (15.45% of total CD8 + cells). At day 7, GFP- positive cells were still detectable in both lymphoid tissues. No GFP- positive cells were detected in mice injected with mock transfected cells.

The suitability of the method described here for the expansion and transfection of mouse strains other than NOD mice was determined using C57Bl/6 mice. Spleen CD8 + cells from C57Bl/6 mice expanded very well, with a 41-fold expansion compared to 18-fold expansion for NOD- derived CD8 + T cells in a parallel experiment. Additionally the expanded C57Bl/6 CD8 + cells transfected with an efficiency comparable to expanded NOD T cells as assessed by GFP expression (90.1% GFP positive for C57Bl/6 compared to 91.7% for NOD CD8 + T cells) in a parallel experiment.

## 4 Discussion

Previous methods for expansion of CD8 + T-cells have used Concanavalin A (Palacios, 1982) and anti-CD3/CD28 beads (Kalamasz et al., 2004); however these methods did not prove reliable for the expansion of murine CD8 + cells of sufficient quantity or viability for *in vivo* immunotherapy experiments in our laboratory. We therefore developed a simple method for the *ex vivo* expansion of CD8 + T cells derived from NOD mouse spleens, activated non-specifically and then supported by cytokines and media supplements summarized in Fig. 1. The cells were initially cultured in plates coated with anti-CD3 and anti-CD28 which result in non-specific stimulation of the T cell receptor and the CD28 co-stimulator for 48 h (supplemented with IL-2 and IL-7 after 24 h), after which they were removed from these plates and sub-cultured in non-coated tissue culture plates in the presence of IL‐2 and IL‐7. When cultured in RPMI 1640 medium containing 10% FCS and supplemented with ITS, this protocol results in a 20-fold expansion of cell number compared to the starting cell number. Supplementing the medium with ITS (insulin, transferrin and selenium) resulted in a significant improvement in expansion. Activation of CD8 + T-cells results in up-regulation of both insulin and IGF-II receptors (Stentz and Kitabchi, 2004). Thus, it is possible that the effect of ITS is due to the high levels of insulin in the ITS (1.7 μM) with insulin acting through the insulin receptor to promote the metabolic changes that occur during T-cell activation, and also causing activation of the IGF-II receptor to promote cell survival (Heaton et al., 1984). We have also shown excellent expansion and high efficiency transfection of C57Bl/6 mouse CD8 + cells indicating that this method is generally applicable to mouse CD8 + cells and not specific to cells derived from the NOD mouse.

The activation of the CD8 + T cells, as shown by upregulation of activation markers and effective expansion of CD8 + T cells allowed *in vivo* tracking in a way that significantly reduced the number of mice used in our research, in keeping with the principle to reduce mouse numbers by refining experimental techniques. Typically, for each experiment where 10^7^ cells were needed for transfer, one donor mouse was required for 8–10 recipient mice. Prior to development of the method reported here, our studies required one donor mouse for each recipient mouse.

In conclusion we have developed a simple and reliable method for large-scale expansion of CD8 + T-cells, which can subsequently be used for transfection and immunotherapy studies.

## Funding

This study was funded by the European Foundation for the Study of Diabetes and the Medical Research Council (UK) grant G0901155.

## Figures and Tables

**Table 1:** Expansion of CD8 + T cells in RPMI 1640 medium with different supplements. Results were compared by analysis of variance (ANOVA) with subsequent comparisons by Student–Newman–Keuls Multiple comparison test.

Medium	Fold expansion after 4 days (mean n = 4)	Standard error	Significance compared to RPMI + 5% FCS	Viability of cells after expansion
RPMI + 5% FCS	5.1	0.95	NA	83.5%
RPMI + 5% FCS + ITS	16.7	3.8	p < 0.01	94.8%
RPMI 10% FCS	13	1.5	p < 0.05	91.8%
RPMI 10% FCS + ITS	20	1.2	p < 0.01	94.7%

**Table 2:** Markers of T-cell activation in expanded and control cells. Expanded and control cells were stained for markers of T-cell activation and analyzed by flow cytometry. Results are expressed as the percentage of live CD8 + cells that are positive for that marker.

	% positive cells	
Marker	Expanded cells	Control cells
CD103	4.7	11.3
CD25	99.6	2.5
LFA-1/CD11a	98.4	31
KLRG1	0	4.5
CTLA-4	13.6	1
CD69	2.1	5.2
CD127/IL-7R	21.2	5
CD215/IL-15Rα	41.1	3.8
Interferon-γ	87.9	14.3
TNF-α	74.2	33.2
IL-10	0	0

**Table 3:** Detection of transfected cells in spleen and PLN. GFP- positive CD8 + T cells (% total CD8 +) at 1, 3 and 7 days in spleen and pancreatic lymph node (PLN) of mice which received 10^7^ GFP-transfected cells on day 0. The background fluorescence from transfer of mock-transfected cells has been subtracted.

	GFP positive CD8 + T cells (% total CD8 +)		
	Day 1	Day 3	Day 7
GFP spleen	6.4	15.11	2.52
GFP PLN	0.32	5.7	1.17
